# Ferroptosis, pyroptosis, and necroptosis in melanoma: regulatory cell death pathways and their implications for immunotherapy

**DOI:** 10.3389/fonc.2026.1820654

**Published:** 2026-05-08

**Authors:** Qingyi Wang, Jian Zhu

**Affiliations:** 1 Department of Dermatology, Qingdao Hospital of University of Health and Rehabilitation Sciences Qingdao Municipal Hospital (Group), Qingdao, Shandong, China; 2 Department of Ophthalmology, Qingdao Hospital of University of Health and Rehabilitation Sciences Qingdao Municipal Hospital (Group), Qingdao, Shandong, China

**Keywords:** ferroptosis, immunotherapy, melanoma, necroptosis, pyroptosis

## Abstract

Melanoma is a highly aggressive skin malignancy with poor prognosis in advanced stages. Despite significant breakthroughs achieved by immune checkpoint inhibitors (ICIs) such as PD-1 and CTLA-4 blockers, challenges including heterogeneous treatment responses, acquired resistance, and immune-related toxicities remain prominent. Ferroptosis, pyroptosis, and necroptosis represent novel forms of regulated cell death (RCD) that have garnered increasing attention due to their capacity to remodel the tumor immune microenvironment (TME) and enhance tumor immunogenicity. These immunogenic cell death (ICD) pathways hold promise as pivotal targets to overcome current limitations in melanoma immunotherapy. This review systematically summarizes the molecular mechanisms by which ferroptosis, pyroptosis, and necroptosis influence melanoma initiation, progression, and response to immunotherapy. We further explore the synergistic interactions between these RCD pathways and ICIs, highlighting their potential to potentiate antitumor immunity. Finally, we discuss emerging therapeutic strategies aimed at selectively inducing these ICD modalities and their translational prospects in clinical melanoma management, offering new avenues to improve patient outcomes.

## 1 Introduction

Melanoma represents the most aggressive and lethal form of skin cancer, characterized by its rapid progression, high metastatic potential, and notable resistance to conventional therapies (1–4). Despite advances in targeted therapies and immune checkpoint inhibitors that have significantly improved survival rates in advanced melanoma, a substantial proportion of patients either fail to respond or develop resistance after initial therapeutic benefit (5, 6). This clinical challenge underscores the urgent need to explore novel mechanisms of melanoma cell death and to identify innovative therapeutic targets that can overcome resistance and improve patient outcomes.

A hallmark of melanoma malignancy is its ability to evade programmed cell death, which contributes to tumor progression and therapeutic resistance (7–9). Traditional apoptotic pathways are frequently impaired in melanoma due to mechanisms such as overexpression of anti-apoptotic Bcl-2 family proteins and constitutive activation of the MAPK signaling pathway (7). Consequently, melanoma cells often exhibit resistance to apoptosis-inducing agents, limiting the efficacy of many conventional treatments. This resistance has prompted increasing interest in alternative regulated cell death (RCD) pathways beyond apoptosis, including ferroptosis, pyroptosis, necroptosis, and more recently described forms such as cuproptosis and triaptosis, which may offer new avenues for therapeutic intervention (10–12).

Ferroptosis, an iron-dependent form of regulated cell death characterized by the accumulation of lipid peroxides and reactive oxygen species (ROS), has emerged as a particularly promising target in melanoma therapy (13–15). Unlike apoptosis, ferroptosis involves distinct morphological and biochemical features, including mitochondrial shrinkage and increased membrane density, and is regulated by key molecules such as glutathione peroxidase 4 (GPX4) and solute carrier family 7 member 11 (SLC7A11) (5, 13, 16). Dysregulation of ferroptosis has been implicated in melanoma progression, with evidence showing that inducing ferroptosis can inhibit tumor growth and sensitize melanoma cells to existing therapies (6, 17). Notably, melanoma cells exhibit heterogeneity in ferroptosis sensitivity, influenced by factors such as metabolic state, gene expression profiles, and microenvironmental conditions, including protection from ferroptosis in the lymphatic system (18, 19). This heterogeneity highlights the complexity of ferroptosis regulation and the need for precise therapeutic strategies.

In addition to ferroptosis, other non-apoptotic RCD pathways such as pyroptosis and necroptosis have gained attention for their roles in melanoma biology and therapy (14, 20). Pyroptosis, mediated by gasdermin proteins and characterized by inflammatory cell death, can enhance antitumor immunity by releasing damage-associated molecular patterns (DAMPs) and pro-inflammatory cytokines, thereby reshaping the tumor microenvironment (TME) (14, 21). Necroptosis, a programmed necrotic cell death pathway governed by the RIPK1/RIPK3/MLKL signaling axis, is subject to sophisticated epigenetic and post-translational regulatory mechanisms in melanoma—epigenetic silencing of core necroptotic genes represents a dominant evasion strategy employed by melanoma cells to suppress this immunogenic cell death modality (7, 22–24). Specifically, hypermethylation of the RIPK3 promoter region and histone deacetylation at the MLKL gene locus are well-documented epigenetic events that abrogate the expression of these key effectors, leading to impaired necrosome assembly and subsequent necroptosis resistance (22, 23, 25). This epigenetic repression is often driven by melanoma-associated epigenetic modifiers, including DNA methyltransferases (DNMT1/DNMT3B) and histone deacetylases (HDAC1/HDAC6), which are frequently overexpressed in advanced melanoma and correlate with poor clinical outcomes (26, 27). Beyond epigenetic regulation, necroptosis is further fine-tuned by caspase-8-mediated cleavage of RIPK1/RIPK3 and post-translational modifications (such as ubiquitination, phosphorylation) of necrosome components, collectively contributing to the dysregulation of this pathway in melanoma (28–30). Necroptosis, regulated by receptor-interacting protein kinases (RIPK1/RIPK3) and mixed lineage kinase domain-like protein (MLKL), similarly contributes to immunogenic cell death and has been associated with improved responses to immunotherapy in melanoma (31, 32). The interplay between these RCD pathways and the immune system is critical, as their activation can convert immunologically “cold” tumors into “hot” tumors, enhancing immune cell infiltration and responsiveness to immune checkpoint blockade (ICB) therapies (7, 14).

The immunogenic nature of ferroptosis, pyroptosis, and necroptosis is particularly relevant in the context of melanoma immunotherapy. Activation of these pathways leads to the release of DAMPs and inflammatory mediators that stimulate both innate and adaptive immune responses, potentially overcoming immune evasion mechanisms employed by melanoma cells (14). For instance, ferroptosis induction has been shown to synergize with ICB by promoting tumor antigen presentation and T cell infiltration, thereby enhancing antitumor immunity (16, 33). Similarly, pyroptosis and necroptosis can potentiate the efficacy of immunotherapies by modulating the TME and facilitating immune cell activation (21, 32). These findings suggest that targeting non-apoptotic RCD pathways may not only directly eliminate melanoma cells but also improve the effectiveness of immunotherapeutic strategies.

Moreover, the molecular regulation of these RCD pathways in melanoma involves complex networks of signaling molecules, transcription factors, and metabolic enzymes. For example, the Wnt/β-catenin-MITF axis has been implicated in modulating ferroptosis sensitivity, with inhibition of this pathway enhancing ferroptosis and improving anti-PD-1 immunotherapy efficacy (34). Other regulators include cytoglobin, which attenuates melanoma malignancy but confers resistance to ferroptosis, and the USP7-JunD-AIFM2 pathway, which inhibits ferroptosis and contributes to therapeutic resistance (35, 36). Understanding these regulatory mechanisms is essential for developing targeted interventions that can modulate RCD pathways to overcome resistance.

The tumor microenvironment and metabolic heterogeneity further influence the susceptibility of melanoma cells to RCD. Metabolic adaptations, such as increased fatty acid oxidation and altered iron metabolism, can protect melanoma cells from ferroptosis and other forms of cell death, contributing to therapy resistance and metastasis (37, 38). Additionally, immune suppressive components within the TME, including regulatory T cells, myeloid-derived suppressor cells, and cancer-associated fibroblasts, interact with melanoma cells to modulate RCD pathways and immune responses (39, 40). Therapeutic strategies that target both melanoma cells and the TME, including the use of nanomaterials for precise drug delivery and combination therapies integrating RCD inducers with immunotherapies, hold promise for improving treatment outcomes (41, 42).

In summary, the malignant progression and therapeutic resistance of melanoma are closely linked to its ability to evade multiple forms of regulated cell death. While traditional apoptosis pathways are often compromised, non-apoptotic RCD pathways such as ferroptosis, pyroptosis, and necroptosis offer novel and promising targets for therapy. Their unique capacity to induce immunogenic cell death and modulate the tumor immune microenvironment positions them at the forefront of emerging strategies to enhance the efficacy of existing treatments, particularly immunotherapies. A comprehensive understanding of the molecular mechanisms governing these RCD pathways, their interplay with melanoma biology and the immune system, and the influence of the tumor microenvironment is critical for the development of innovative combination therapies. Such approaches have the potential to overcome resistance, convert immunologically “cold” tumors into “hot,” and ultimately improve the prognosis for patients with melanoma. This review systematically elucidates the molecular mechanisms, immune regulatory functions, and therapeutic implications of ferroptosis, pyroptosis, and necroptosis in melanoma, aiming to provide a foundation for future research and clinical translation.

## 2 Molecular mechanisms of ferroptosis in melanoma and its immunomodulatory functions

Melanoma is one of the most aggressive cutaneous malignancies with high metastatic potential and poor prognosis, especially in advanced stages where conventional therapies and immunotherapies often face limitations due to intrinsic or acquired resistance (14, 16, 43, 44). Ferroptosis, as a newly identified form of regulated cell death, has emerged as a critical regulatory node in melanoma progression, therapy resistance, and immune response modulation (13, 14, 16). Accumulating evidence indicates that the dysregulation of ferroptosis-related biochemical pathways is closely associated with melanoma initiation and progression, while ferroptosis-induced immunogenic cell death (ICD) can profoundly reshape the tumor microenvironment (TME) and influence anti-tumor immune responses (45–48). This section will systematically elaborate on the core biochemical pathways governing ferroptosis in melanoma, the regulatory mechanisms underlying ferroptosis resistance, and the intricate interplay between ferroptosis and immune modulation, aiming to provide a comprehensive understanding of ferroptosis as a potential therapeutic target for melanoma.

### 2.1 Core biochemical pathways of ferroptosis and their regulation in melanoma

Ferroptosis is a distinct form of regulated cell death characterized by iron-dependent lipid peroxidation (LPO), fundamentally driven by the loss of activity of glutathione peroxidase 4 (GPX4), which normally detoxifies lipid hydroperoxides (49–51). In melanoma, the core biochemical pathway of ferroptosis revolves around the accumulation of iron and subsequent peroxidation of polyunsaturated fatty acids (PUFAs) in cellular membranes, culminating in lethal oxidative damage (13, 14, 19, 52). The oncogenic BRAF V600E mutation, prevalent in melanoma, exerts a pivotal role in ferroptosis regulation by upregulating SLC7A11, the light chain subunit of system Xc-, which imports cystine for glutathione (GSH) synthesis. Enhanced cystine uptake and GSH biosynthesis bolster GPX4 activity, thereby suppressing ferroptosis and promoting tumor growth and resistance to therapy (5, 6, 53). This metabolic adaptation underscores a critical survival mechanism in melanoma cells, enabling evasion of ferroptotic cell death.

Melanoma cells exhibit remarkable metabolic plasticity to resist ferroptosis. Activation of the NRF2 pathway, a master regulator of antioxidant responses, leads to upregulation of iron storage proteins such as ferritin heavy chain 1 (FTH1), which sequesters labile iron, reducing the pool of redox-active iron that catalyzes lipid peroxidation (34, 54). Additionally, melanoma cells reprogram lipid metabolism by increasing the synthesis of monounsaturated fatty acids (MUFAs) via stearoyl-CoA desaturase (SCD), which replace PUFAs in membrane phospholipids, thereby reducing susceptibility to lipid peroxidation and ferroptosis (55). This lipid remodeling confers a density-dependent ferroptosis resistance, as higher cell density induces SCD expression and a lipogenic phenotype. Furthermore, secreted apolipoprotein E (ApoE) from melanoma cells modulates ferroptosis sensitivity by decreasing peroxidation-prone PUFA content and enhancing GPX4 levels, contributing to ferroptosis resistance and correlating with poor patient prognosis (19).

**Figure 1:**
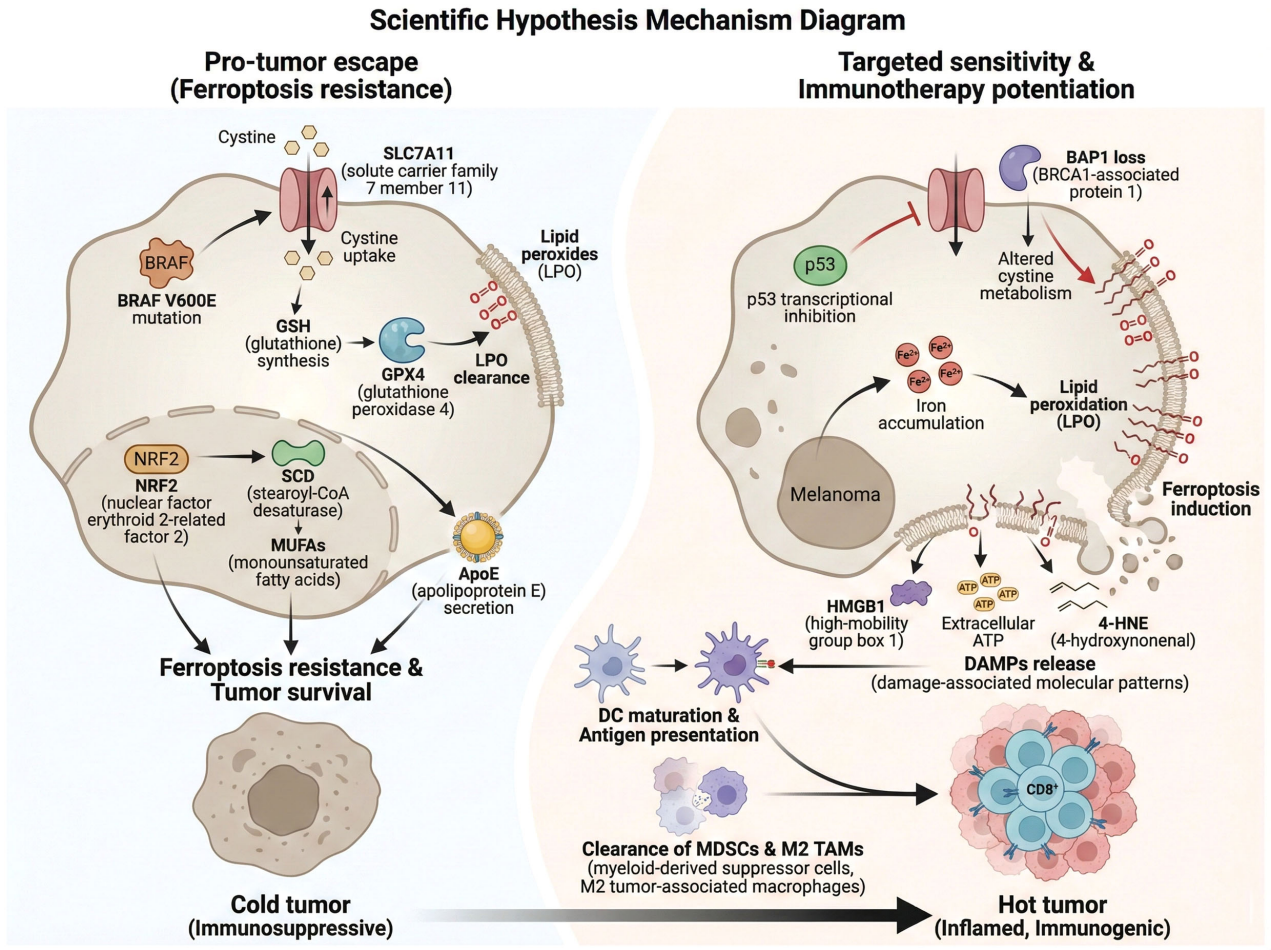
Schematically illustrates the dual role of ferroptosis modulation in melanoma, including the molecular mechanisms of ferroptosis resistance that drive tumor immune escape and the ferroptosis-induced immunogenic cell death that remodels the tumor microenvironment to potentiate immunotherapy efficacy.

Tumor suppressor genes such as p53 and BAP1 also intricately regulate ferroptosis sensitivity in melanoma. p53 can transcriptionally repress SLC7A11, thereby sensitizing cells to ferroptosis; however, mutations or loss of p53 disrupt this regulation, leading to ferroptosis resistance (53). Similarly, BAP1 loss impairs ferroptosis by modulating cystine uptake and lipid metabolism, further complicating the ferroptosis regulatory network. The complexity of these interactions highlights the multifaceted control of ferroptosis in melanoma, where genetic alterations, metabolic reprogramming, and antioxidant defenses converge to determine cell fate. Targeting these pathways, such as inhibiting SLC7A11 or SCD, or modulating NRF2 activity, represents promising therapeutic strategies to overcome ferroptosis resistance and improve melanoma treatment outcomes (**Figure 1**) (16, 56, 57).

### 2.2 Immunogenicity induced by ferroptosis and remodeling of the tumor microenvironment

Ferroptosis in melanoma not only induces tumor cell death but also profoundly influences the tumor microenvironment (TME) through the release of damage-associated molecular patterns (DAMPs) and lipid peroxidation products, which modulate immune responses (45, 47). Ferroptotic melanoma cells release high-mobility group box 1 (HMGB1), extracellular ATP, and lipid peroxidation end-products such as 4-hydroxynonenal (4-HNE), which serve as potent DAMPs (58–60). These molecules engage pattern recognition receptors, including Toll-like receptors (TLRs) on dendritic cells (DCs), promoting DC maturation and antigen presentation, thereby enhancing anti-tumor immunity (14). This immunogenic cell death (ICD) phenotype of ferroptosis contrasts with immunologically silent apoptosis and underscores ferroptosis as a bridge between tumor cell death and immune activation.

Moreover, ferroptosis selectively depletes immunosuppressive cells within the TME, such as myeloid-derived suppressor cells (MDSCs) and M2-polarized tumor-associated macrophages (TAMs), which are particularly vulnerable to lipid peroxidation due to their metabolic profiles (14, 59, 61). The reduction of these suppressive populations alleviates immune inhibition, facilitating the infiltration and activation of cytotoxic CD8+ T cells (45, 62). Ferroptosis-derived oxidized phospholipids and other lipid mediators further promote CD8+ T cell recruitment and effector functions, enhancing anti-melanoma immunity (14, 46, 63). However, this process is double-edged; certain oxidized lipid species generated during ferroptosis may exert immunosuppressive effects, potentially dampening T cell responses and contributing to immune evasion (43, 64). This duality necessitates precise modulation of ferroptosis to maximize immunostimulatory effects while minimizing immunosuppression (65).

The interplay between ferroptosis and immune modulation also impacts the efficacy of immunotherapies. Induction of ferroptosis can synergize with immune checkpoint inhibitors (ICIs) by reshaping the TME towards a more immunogenic state, overcoming resistance mechanisms (14, 20, 45). Conversely, metabolic adaptations in melanoma cells and immune cells within the TME, such as upregulation of antioxidant pathways and lipid metabolism reprogramming, can attenuate ferroptosis-induced immunogenicity (20, 33). Therefore, combinatorial strategies targeting ferroptosis pathways alongside immunotherapies hold promise for enhancing therapeutic responses in melanoma (66, 67). Understanding the molecular signals released during ferroptosis and their effects on various immune subsets is critical for designing interventions that harness ferroptosis to remodel the TME favorably and potentiate anti-tumor immunity (14, 60).

## 3 Activation pathways of pyroptosis in melanoma and its pro-inflammatory effects

Pyroptosis, as a unique form of regulated inflammatory cell death, has emerged as a pivotal regulatory node in the progression of melanoma and the efficacy of antitumor therapies (68, 69). Unlike other forms of cell death, pyroptosis is characterized by its intrinsic pro-inflammatory properties, which not only mediate direct tumoricidal effects but also actively modulate the tumor immune microenvironment, thereby influencing the antitumor immune response (7, 70). In recent years, accumulating evidence has uncovered the complex activation pathways of pyroptosis in melanoma cells and clarified its critical role in linking cell death to inflammatory signaling and immune activation (14). This section will systematically elaborate on the core molecular mechanisms underlying pyroptosis activation in melanoma, focusing on the gasdermin protein-mediated execution process and the pro-inflammatory effects associated with pyroptosis, while discussing the implications of these pathways for the development of novel melanoma therapeutic strategies.

### 3.1 Gasdermin protein-mediated execution mechanism of pyroptosis

**Figure 2:**
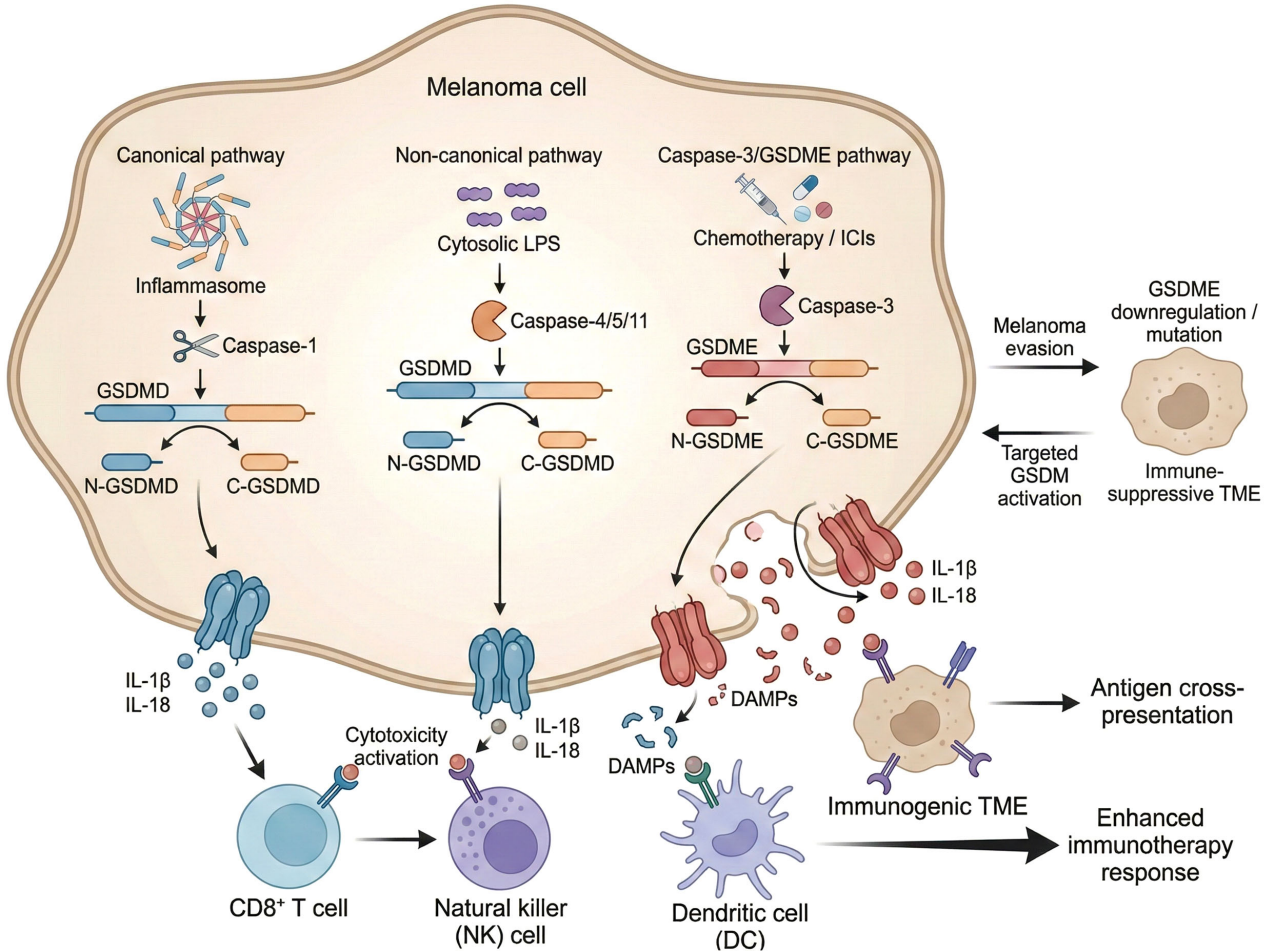
Schematically depicts the three major pyroptosis pathways in melanoma cells (canonical, non-canonical, and caspase-3/GSDME pathways) and their downstream immunomodulatory effects. Activation of these pathways leads to the release of pro-inflammatory cytokines and DAMPs, which activate CD8+ T cells, NK cells, and dendritic cells, thereby remodeling the tumor microenvironment and enhancing anti-melanoma immunotherapy responses.

Pyroptosis is a form of regulated inflammatory cell death primarily executed by the gasdermin (GSDM) family of proteins, which includes members such as GSDMD and GSDME (71, 72). These proteins share a conserved N-terminal domain capable of forming membrane pores that disrupt cellular integrity, leading to cell swelling, membrane rupture, and release of intracellular contents (73). The activation of pyroptosis involves proteolytic cleavage of gasdermins by specific caspases, which liberates the pore-forming N-terminal fragment from the autoinhibitory C-terminal domain (74). In the canonical pyroptosis pathway, caspase-1 cleaves GSDMD, whereas the noncanonical pathway involves caspase-4/5 in humans or caspase-11 in mice cleaving GSDMD (8, 20). Notably, in melanoma cells, pyroptosis can also be induced via caspase-3-mediated cleavage of GSDME, linking apoptotic stimuli to pyroptotic execution (75–77). Chemotherapeutic agents and immune checkpoint inhibitors (ICIs) have been shown to activate caspase-3, which subsequently cleaves GSDME, triggering pyroptosis in melanoma cells (75, 78). This caspase-3/GSDME axis represents a critical mechanism by which melanoma cells undergo inflammatory cell death in response to therapy, potentially enhancing antitumor immunity (21, 79, 80). However, melanoma cells may evade pyroptosis by downregulating the expression of gasdermin proteins or acquiring mutations that impair their function, contributing to immune escape and resistance to therapy (8, 81). For instance, reduced GSDME expression or loss-of-function mutations can diminish pyroptotic responses, allowing melanoma cells to survive cytotoxic insults and evade immune surveillance (77, 82). This evasion mechanism underscores the importance of gasdermin expression levels and integrity in determining the susceptibility of melanoma cells to pyroptosis and highlights the potential of targeting gasdermin pathways to overcome immune resistance (21, 82). Overall, the gasdermin-mediated pore formation is a pivotal step in pyroptosis execution, integrating signals from classical inflammasome pathways and apoptotic caspases, and its modulation in melanoma has significant implications for therapeutic strategies aimed at inducing immunogenic cell death (**Figure 2**) (71, 72).

### 3.2 Pyroptosis-associated inflammatory cytokine release and antitumor immune activation

The formation of gasdermin pores during pyroptosis leads to a characteristic cellular swelling and eventual rupture of the plasma membrane, resulting in the release of pro-inflammatory cytokines such as interleukin-1β (IL-1β) and interleukin-18 (IL-18) (72, 83). These cytokines are central to the initiation and amplification of antitumor immune responses. IL-1β acts as a potent activator of CD8+ cytotoxic T lymphocytes and natural killer (NK) cells, promoting their proliferation and enhancing their cytotoxic functions against tumor cells (69). Concurrently, IL-18 synergizes with IL-1β to augment the cytotoxic capacity of effector T cells and stimulate the production of interferon-gamma (IFN-γ), a key cytokine driving Th1-type immune responses that are critical for effective tumor clearance. The robust inflammatory milieu generated by pyroptosis-induced cytokine release can reverse the immunosuppressive tumor microenvironment characteristic of melanoma, facilitating increased infiltration and activation of tumor-infiltrating lymphocytes (TILs) (20, 82). This shift in the tumor immune landscape creates a more favorable context for immune checkpoint inhibitors (ICIs) to exert their therapeutic effects, as the enhanced immune activation overcomes prior resistance mechanisms (21, 82, 84). Moreover, the release of damage-associated molecular patterns (DAMPs) through gasdermin pores further stimulates antigen-presenting cells, promoting antigen cross-presentation and the priming of adaptive immune responses. This cascade of events underscores the dual role of pyroptosis as both a direct tumoricidal mechanism and an immunogenic trigger that potentiates systemic antitumor immunity (14). Therapeutic approaches that induce pyroptosis in melanoma, such as chemotherapeutics, metabolic inhibitors, or novel agents targeting gasdermin activation, have demonstrated enhanced immune cell infiltration and improved responses to immunotherapy in preclinical models (60, 78). Thus, harnessing pyroptosis-mediated inflammatory signaling represents a promising strategy to overcome melanoma immune evasion and improve clinical outcomes (21, 82, 85).

## 4 Signal transduction of necroptosis and its role in immune memory in melanoma

Melanoma, a highly aggressive cutaneous malignancy characterized by remarkable heterogeneity and immune evasion capacity, remains a major clinical challenge despite advances in immunotherapeutic strategies (8, 86). Necroptosis, as a programmed necrotic cell death pathway distinct from apoptosis and accidental necrosis, has emerged as a key regulator of anti-tumor immunity, particularly in shaping immune memory responses that are critical for long-term tumor control (87, 88). Accumulating evidence indicates that the dysregulation of necroptotic signaling cascades contributes to melanoma progression, while targeted modulation of this pathway holds promise for enhancing the efficacy of immunotherapies (86). This section will first elaborate on the core molecular mechanisms underlying necroptosis, focusing on the RIPK1/RIPK3/MLKL signaling axis and necrosome assembly, followed by a discussion of how necroptosis modulates antigen cross-presentation and the generation of durable immune memory in the context of melanoma.

### 4.1 RIPK1/RIPK3/MLKL signaling axis and necrosome formation

Necroptosis, a regulated form of necrotic cell death, is orchestrated by a signaling cascade involving receptor-interacting protein kinase 1 (RIPK1), RIPK3, and mixed lineage kinase domain-like protein (MLKL) (89, 90). This pathway is typically activated downstream of death receptors such as tumor necrosis factor receptor 1 (TNFR1) upon binding of ligands like tumor necrosis factor-alpha (TNF-α), especially when apoptotic pathways are inhibited, for example, through caspase-8 inactivation (91). Under these conditions, RIPK1 interacts with RIPK3 via their RIP homotypic interaction motifs (RHIM), leading to the assembly of a high molecular weight complex termed the necrosome (92). Within this complex, RIPK3 phosphorylates MLKL, triggering MLKL oligomerization and translocation to the plasma membrane, where it disrupts membrane integrity to execute necroptotic cell death (93–95). The formation and function of the necrosome are tightly regulated by post-translational modifications such as phosphorylation, ubiquitination, and proteolytic cleavage, which modulate the stability, activity, and interactions of RIPK1, RIPK3, and MLKL, thereby fine-tuning necroptotic signaling (94, 96). Recent studies have also highlighted the involvement of additional regulatory proteins, including casein kinase 1 (CK1) family members, which phosphorylate RIPK3 at serine 227, a critical step for MLKL recruitment and activation (95). Furthermore, the necrosome has been shown to localize near the endoplasmic reticulum (ER), where activated MLKL can initiate ER stress responses, linking necroptosis to broader cellular stress pathways (97).

In the context of melanoma, the necroptotic pathway is frequently dysregulated. Epigenetic modifications constitute the primary and most well-characterized mechanism of necroptosis suppression in melanoma, with RIPK3 serving as the major epigenetic target (22, 23, 98). Genome-wide methylation profiling of melanoma tissues and cell lines has demonstrated that the RIPK3 promoter region exhibits significantly higher CpG island hypermethylation in metastatic melanoma compared to primary lesions and normal melanocytes, a change that directly correlates with reduced RIPK3 mRNA and protein expression (99). This hypermethylation is driven by the upregulation of DNMT1 and DNMT3B in melanoma cells, DNMT1 mediates the maintenance of DNA methylation patterns during cell division, while DNMT3B promotes *de novo* methylation of the RIPK3 promoter, leading to stable and heritable silencing of the gene (27, 98). In addition to DNA methylation, histone modifications further reinforce necroptosis repression: HDAC1 and HDAC6 bind to the MLKL and RIPK3 gene loci, removing acetyl groups from histone H3 and H4, which results in chromatin condensation and impaired transcription factor binding (such as NF-κB, AP-1) required for gene expression (22, 100). Pharmacological inhibition of epigenetic modifiers—such as DNMT inhibitors (5-azacytidine) and HDAC inhibitors (vorinostat)—has been shown to reverse RIPK3 and MLKL silencing in melanoma cell lines, restoring necrosome formation and sensitizing cells to TNF-α-induced necroptosis (98, 101, 102). One key mechanism involves the epigenetic silencing of RIPK3 expression, which diminishes necrosome formation and necroptotic execution, thereby promoting tumor cell survival and resistance to cell death (7, 14). Additionally, elevated caspase-8 activity in melanoma cells can inhibit necroptosis by cleaving RIPK1 and RIPK3, preventing necrosome assembly (103). This suppression of necroptosis contributes to melanoma progression by allowing malignant cells to evade immunogenic cell death pathways. Experimental evidence from other cancer models, including breast cancer and melanoma cell lines, demonstrates that pharmacological or genetic inhibition of RIPK1, RIPK3, or MLKL can modulate necroptotic cell death, underscoring the therapeutic potential of targeting this axis (104). Moreover, natural compounds such as andrographolide have been shown to inhibit the RIPK1/RIPK3/MLKL pathway by reducing reactive oxygen species (ROS) generation and preserving mitochondrial integrity, further illustrating the complex interplay between necroptotic signaling and cellular metabolism (105).

**Figure 3:**
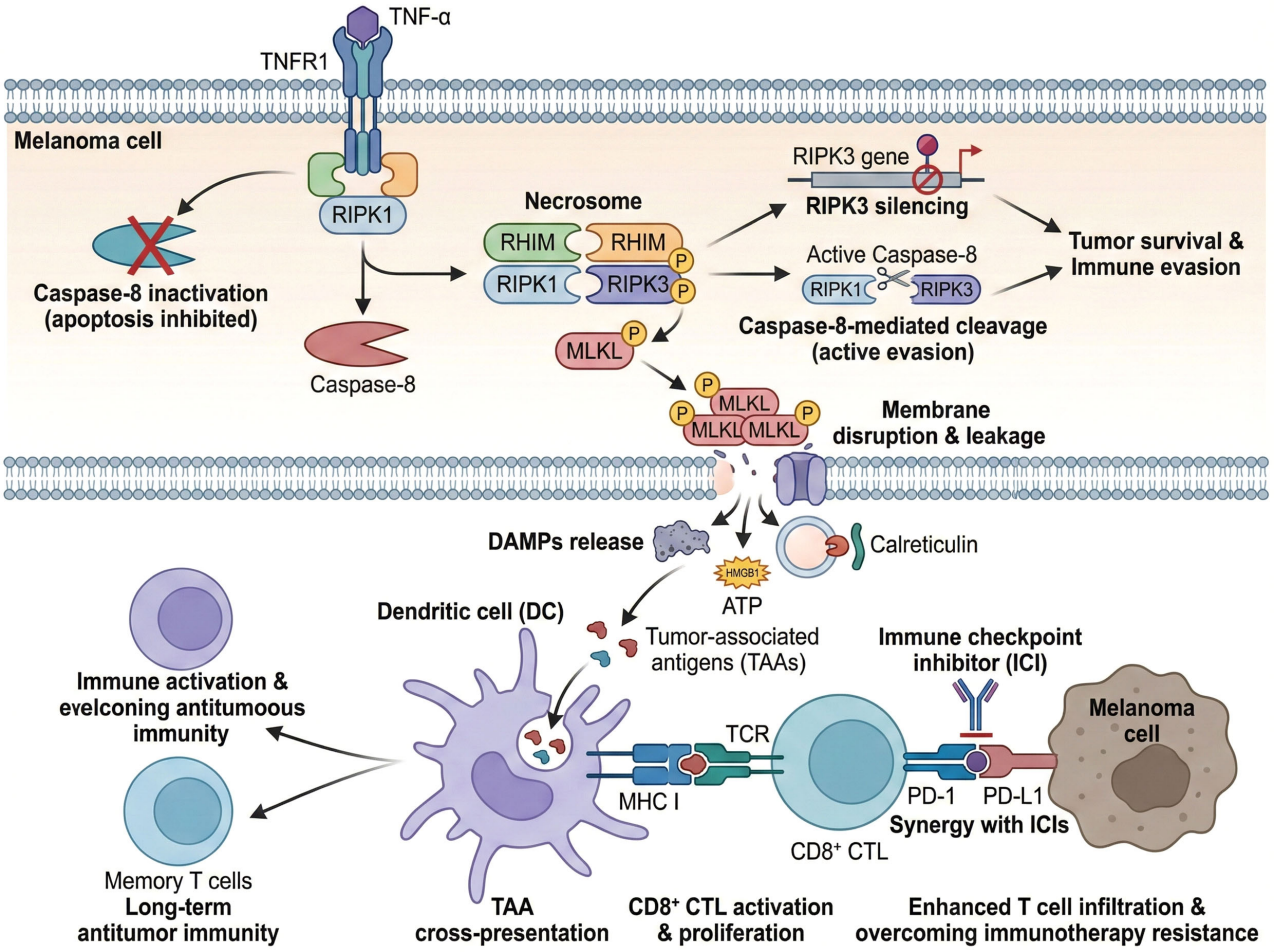
Schematically depicts the RIPK1/RIPK3/MLKL signaling axis and necrosome formation in melanoma, as well as the mechanisms of tumor evasion and downstream immunomodulatory effects. It illustrates the activation of necroptosis upon TNF-α stimulation and caspase-8 inactivation, the suppression of this pathway in melanoma via epigenetic silencing of RIPK3 or caspase-8-mediated cleavage, and the release of DAMPs following necroptosis that promotes dendritic cell activation and antigen cross-presentation.

Collectively, the RIPK1/RIPK3/MLKL signaling axis serves as a central regulatory hub for necroptosis, with necrosome formation being a critical step in executing this form of programmed necrosis. In melanoma, the frequent suppression of this pathway through epigenetic and enzymatic mechanisms facilitates tumor cell survival and immune evasion, highlighting the importance of understanding and potentially restoring necroptotic signaling for therapeutic benefit (**Figure 3**).

### 4.2 Necroptosis promotes antigen cross-presentation and durable immunity

Restoration of necroptotic signaling in melanoma, either through epigenetic modifier inhibitors to reverse RIPK3/MLKL silencing or direct small-molecule activation of the RIPK1/RIPK3/MLKL axis, enhances tumor cell immunogenicity by amplifying DAMP release and antigen cross-presentation, a phenomenon that has been validated in both *in vitro* melanoma cell models and *in vivo* syngeneic melanoma mouse models (24, 106). Necroptotic cell death is inherently pro-inflammatory and immunogenic due to the release of damage-associated molecular patterns (DAMPs) and tumor-associated antigens (TAAs) upon plasma membrane rupture (107). In melanoma, cells undergoing necroptosis release a spectrum of DAMPs, including high mobility group box 1 (HMGB1), ATP, and calreticulin, which serve as potent activators of dendritic cells (DCs). These activated DCs efficiently uptake necroptotic melanoma cell debris and process tumor antigens for cross-presentation via major histocompatibility complex class I (MHC I) molecules to CD8+ cytotoxic T lymphocytes (CTLs) (7, 14). This cross-priming is critical for initiating robust adaptive immune responses against tumor neoantigens, which are often poorly immunogenic in the context of apoptotic or non-immunogenic cell death (108). The necroptotic pathway thus bridges innate and adaptive immunity by converting dying tumor cells into an endogenous vaccine that stimulates CTL activation.

Importantly, necroptosis-induced antigen cross-presentation not only enhances the initial anti-tumor immune response but also contributes to the generation of memory T cells, which provide long-lasting immune surveillance and protection against tumor recurrence (14). This durable immunity is particularly relevant in melanoma, where immune checkpoint blockade therapies targeting CTLA-4 and PD-1/PD-L1 have revolutionized treatment but are limited by heterogeneous response rates and acquired resistance. Combining necroptosis induction with immune checkpoint inhibitors (ICIs) has shown synergistic effects in preclinical models, enhancing T cell infiltration, cytokine production, and tumor regression (14). For necroptosis-based combination therapy, the triple regimen of epigenetic modifier inhibitors (DNMT/HDAC inhibitors), SMAC mimetics (necroptosis activators), and anti-PD-L1/PD-1 antibodies has entered early-phase clinical trials for advanced metastatic melanoma: this combination reverses epigenetic silencing of core necroptotic genes, relieves caspase-8-mediated enzymatic inhibition of the necrosome, and enhances the anti-tumor cytotoxic T lymphocyte (CTL) response, with preliminary clinical data showing improved objective response rates (ORR) in RIPK3-low melanoma patients who are typically refractory to single-agent ICI therapy (22, 109–111). For example, pharmacological agents or natural compounds that activate the RIPK1/RIPK3/MLKL axis can sensitize melanoma cells to necroptosis, thereby increasing tumor immunogenicity and improving responsiveness to ICIs (7).

Furthermore, necroptosis-associated DAMPs can modulate the tumor microenvironment (TME) by recruiting and activating various immune effector cells, including natural killer (NK) cells and macrophages, which further amplify anti-tumor immunity (7). The inflammatory milieu generated by necroptotic cell death also promotes the maturation and antigen-presenting capacity of DCs, facilitating a positive feedback loop that sustains immune activation. However, the balance of necroptosis must be carefully regulated, as excessive or chronic necroptosis can lead to pathological inflammation and immune suppression, underscoring the need for precise therapeutic modulation.

In summary, necroptosis in melanoma serves as a potent immunogenic cell death modality that enhances antigen cross-presentation by DCs and fosters durable CD8+ T cell-mediated immunity. The integration of necroptosis-inducing strategies with immunotherapies holds promise for overcoming immune resistance and achieving sustained tumor control, representing a compelling avenue for future clinical translation.

### 4.3 Ferroptosis, pyroptosis, and necroptosis in melanoma: similarities, differences, and interplay

Ferroptosis, pyroptosis, and necroptosis are distinct yet interconnected RCD pathways that play pivotal roles in melanoma biology and immunotherapy response, with shared immunogenic properties but divergent molecular mechanisms and functional outputs (7, 14, 112). Clarifying their commonalities, disparities, and crosstalk provides a foundation for optimizing combinatorial therapeutic strategies. All three pathways qualify as immunogenic cell death (ICD) modalities, characterized by the release of damage-associated molecular patterns (DAMPs) such as HMGB1, ATP, and calreticulin upon cell death (10, 14, 112). These DAMPs activate dendritic cells, promote antigen presentation, and bridge innate and adaptive immunity, converting immunologically “cold” melanoma tumors into “hot” microenvironments that enhance T cell infiltration and responsiveness to immune checkpoint inhibitors (ICIs) (7, 14, 113). Functionally, each pathway circumvents melanoma’s intrinsic apoptosis resistance, and their dysregulation—via genetic mutations, epigenetic silencing, or metabolic reprogramming—contributes to tumor progression and therapeutic resistance, highlighting their shared role as tumor-suppressive mechanisms when functionally intact (7, 112, 114).

Despite these commonalities, the pathways diverge sharply in their molecular machinery and biochemical hallmarks. Ferroptosis is an iron-dependent process driven by lipid peroxidation, governed by the inactivation of glutathione peroxidase 4 (GPX4) and dysregulation of iron metabolism (such as SLC7A11/GSH axis, NRF2-FTH1 pathway), with morphological features including mitochondrial shrinkage and membrane lipid peroxidation independent of caspase activation (11, 115, 116). Pyroptosis is mediated exclusively by gasdermin family proteins (GSDMD, GSDME), whose cleavage by caspases (caspase-1 for canonical, caspase-4/5/11 for noncanonical, caspase-3 for GSDME-dependent pathways) forms plasma membrane pores, leading to cellular swelling, rupture, and release of pro-inflammatory cytokines (IL-1β, IL-18)—a defining feature that makes pyroptosis the most potently inflammatory of the three pathways (14, 117). Necroptosis, in contrast, is orchestrated by the RIPK1/RIPK3/MLKL signaling axis and necrosome assembly, triggered by death receptor activation (e.g., TNFR1-TNF-α) and apoptotic pathway inhibition (e.g., caspase-8 inactivation), with execution involving MLKL-mediated plasma membrane disruption and a necrotic morphological phenotype (14, 118). Clinically, these differences translate to variable prognostic implications: elevated GSDME or RIPK3 expression correlates with improved ICI responses, while high GPX4 levels are associated with treatment resistance (75, 119).

The three pathways exhibit intricate crosstalk in melanoma, often functioning synergistically or compensatorily (120). Reactive oxygen species (ROS) serves as a central signaling hub: ferroptosis-induced lipid peroxidation can amplify pyroptosis by activating inflammasomes, while necroptosis-associated ROS production enhances ferroptosis sensitivity via iron release (14, 121). For instance, inhibition of GPX4 increases lipid peroxides that prime caspase-1-dependent GSDMD cleavage, augmenting pyroptotic inflammation (14, 46). Conversely, pyroptosis-derived IL-1β can upregulate RIPK3 expression, sensitizing melanoma cells to necroptosis (7). Metabolic pathways also link these processes: the NRF2 pathway, a key regulator of ferroptosis resistance, concurrently modulates pyroptosis by suppressing ROS-dependent inflammasome activation (11, 122). Notably, compensatory crosstalk may limit monotherapy efficacy—melanoma cells escaping ferroptosis via FSP1 upregulation may become more dependent on necroptosis suppression, and vice versa—underscoring the rationale for co-targeting multiple pathways to avoid adaptive resistance (114).

## 5 Synergistic therapeutic strategies targeting RCD pathways and immune checkpoint inhibitors

Regulated cell death (RCD) pathways, including ferroptosis, pyroptosis, and necroptosis, have emerged as crucial modulators of antitumor immunity, serving as a bridge between tumor cell elimination and immune response activation (14, 123). Immune checkpoint inhibitors (ICIs) have revolutionized melanoma treatment by unleashing antitumor T cell responses, but their clinical efficacy is often limited by primary or acquired resistance, as well as inadequate tumor immunogenicity (14, 123–125). To address these bottlenecks, synergistic therapeutic strategies integrating RCD pathway modulation with ICI-based immunotherapy have gained increasing attention in preclinical and early clinical studies. These combination approaches aim to enhance tumor immunogenicity through targeted RCD induction, overcome immune evasion mechanisms, and improve the depth and durability of antitumor responses (14). Below, we summarize the key advances in three major directions of such synergistic strategies: pharmacological RCD inducers combined with ICIs, nanotechnology-driven precision delivery systems, and strategies to address critical challenges hindering clinical translation, including tumor metabolic plasticity and off-target toxicities.

### 5.1 pharmacological inducers combined with immune checkpoint inhibitors

The combination of pharmacological inducers of regulated cell death (RCD), particularly ferroptosis inducers such as RSL3, Erastin, and GPX4 inhibitors, with immune checkpoint inhibitors (ICIs) like anti-PD-1 antibodies, has demonstrated promising synergistic antitumor effects in preclinical melanoma models (14). Ferroptosis, an iron-dependent form of lipid peroxidation-driven cell death, can sensitize melanoma cells to immune-mediated killing and overcome both primary and acquired resistance to ICIs (126). For instance, the induction of ferroptosis disrupts tumor cell redox homeostasis and promotes immunogenic cell death, thereby enhancing T cell infiltration and activation within the tumor microenvironment (14). This synergism is critical because resistance to ICIs remains a major clinical challenge in melanoma treatment, often due to tumor cell dedifferentiation and immune evasion mechanisms (127). Beyond direct ferroptosis induction, activation of innate immune pathways such as the cGAS-STING axis has been shown to potentiate ICI efficacy (128). Certain chemotherapeutic agents and targeted therapies, including BRAF inhibitors, can indirectly induce pyroptosis or necroptosis—forms of immunogenic cell death—thereby amplifying antitumor immune responses when combined with ICIs. For example, BRAF inhibitors not only suppress oncogenic signaling but also trigger multiple RCD pathways in melanoma cells, which may enhance antigen presentation and T cell priming (129). These findings underscore the therapeutic potential of integrating RCD inducers with ICIs to overcome immunotherapy resistance and improve durable responses in melanoma patients (7, 130–132). Moreover, recent studies have identified novel molecular targets such as ULK1 and PRMT5 that modulate immune resistance mechanisms and can be pharmacologically inhibited to synergize with ICIs, further supporting the rationale for combination strategies (133, 134). Collectively, these data advocate for the clinical development of combination regimens that harness ferroptosis and other RCD pathways alongside immune checkpoint blockade to enhance antitumor immunity and overcome therapeutic resistance in melanoma.

### 5.2 Nanotechnology-driven precision delivery systems

Nanotechnology-based delivery platforms have emerged as a transformative approach to enhance the therapeutic index of combined RCD inducers and ICIs in melanoma treatment (135–137). A variety of structurally distinct nanosystems have been explored for this purpose, including lipid-based nanoparticles, polymeric biodegradable nanoparticles, mesoporous silica nanocarriers, metal–organic frameworks, and stimuli-responsive nanogels (138–140). These nanosystems exhibit favorable drug-loading performance and controlled release characteristics tailored to the physicochemical properties of RCD modulators and immune checkpoint inhibitors (141–143). Nanoparticle systems enable the co-delivery of ferroptosis inducers—such as GPX4 inhibitors—and immune checkpoint blockade agents like anti-PD-L1 antibodies directly to the tumor site, thereby increasing local drug concentration while minimizing systemic toxicity (144, 145). These nanoscale carriers can be engineered to respond to specific tumor microenvironmental cues, including acidic pH, elevated reactive oxygen species (ROS), or tumor-associated enzymes, facilitating controlled and targeted drug release (146, 147). For instance, pH-sensitive or ROS-responsive nanocarriers can selectively release their payload within the melanoma microenvironment, precisely inducing immunogenic cell death modalities such as ferroptosis or pyroptosis in tumor cells. Preclinical investigations have validated the favorable biosafety profiles of these nanodelivery systems in melanoma models, with negligible systemic toxicity and off-target tissue effects (136, 148–152). This targeted induction of RCD not only promotes tumor cell elimination but also enhances antigen release and presentation, thereby potentiating the efficacy of ICIs (153, 154). Additionally, nanocarriers can protect labile therapeutic agents from premature degradation and improve their pharmacokinetics and biodistribution (155). The integration of nanotechnology with immunotherapy thus represents a promising strategy to overcome the limitations of conventional systemic administration, including off-target effects and suboptimal tumor penetration (136, 137). Recent advances have demonstrated that such responsive nanodelivery systems can effectively modulate the tumor immune microenvironment, augment T cell infiltration, and synergize with ICIs to achieve superior antitumor responses in melanoma models (7, 132). Future development of multifunctional nanoplatforms capable of co-delivering multiple RCD inducers and immunomodulators holds great potential to refine precision oncology approaches for melanoma, enabling personalized and highly effective combination therapies (137).

### 5.3 Overcoming challenges in combination therapy: metabolic plasticity and off-target effects

**Figure 4:**
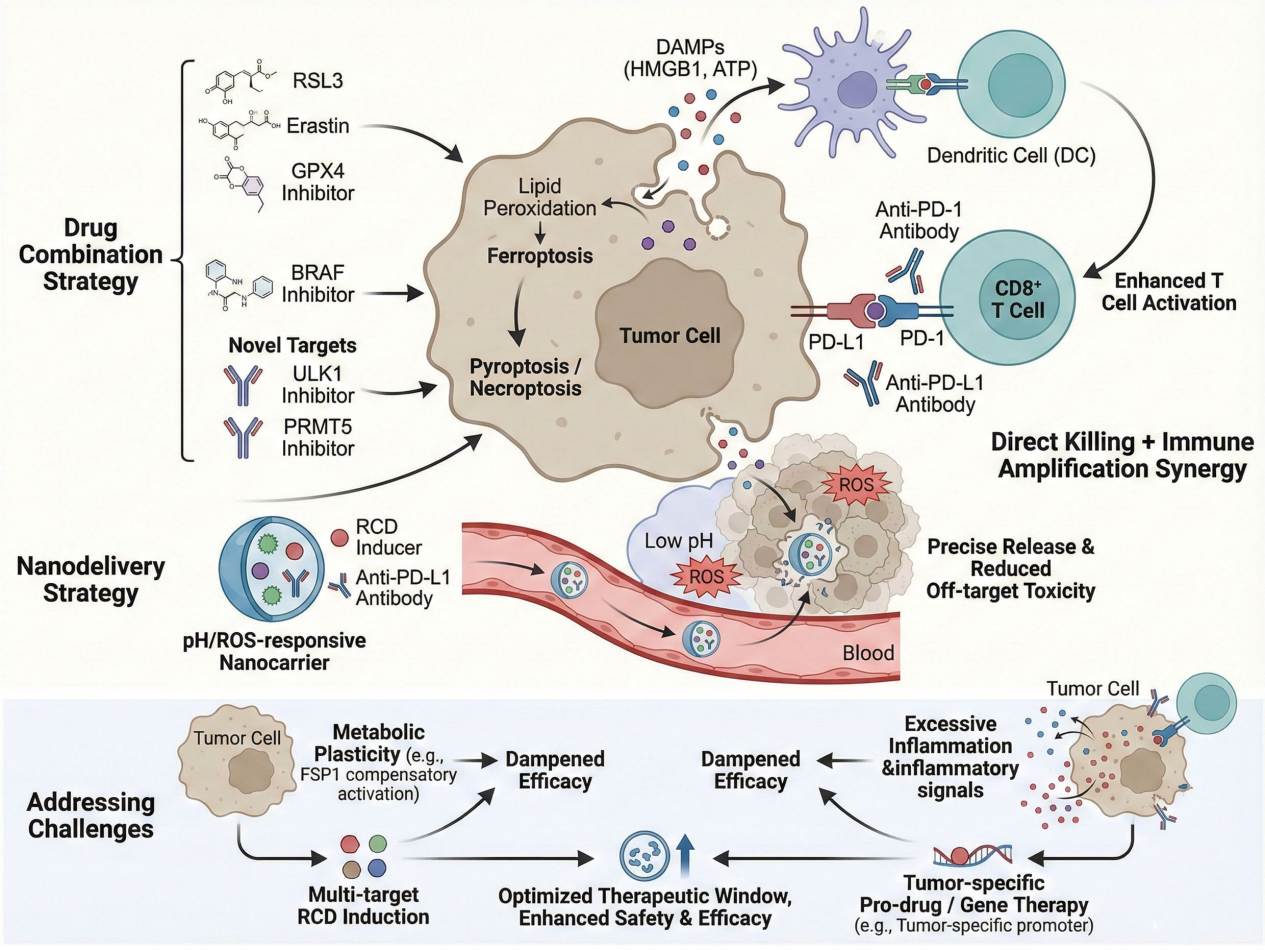
Schematically illustrates the integrated therapeutic strategies targeting regulated cell death (RCD) in melanoma, including drug combinations, nanodelivery systems, and approaches to address key therapeutic challenges. It depicts how the induction of ferroptosis, pyroptosis, or necroptosis can be combined with immune checkpoint inhibitors to achieve direct tumor killing and immune amplification, and how nanocarriers and multi-targeted approaches can overcome limitations such as off-target toxicity and metabolic plasticity to enhance the safety and efficacy of RCD-based therapies.

Despite the promising therapeutic potential of combining RCD inducers with ICIs, several challenges impede clinical translation, notably tumor metabolic plasticity and off-target toxicities (156, 157). Melanoma cells exhibit remarkable metabolic adaptability, which can undermine the efficacy of ferroptosis induction (158). For example, melanoma cells may shift from a GPX4-dependent ferroptosis resistance mechanism to reliance on alternative pathways such as FSP1-mediated lipid peroxide detoxification, thereby evading ferroptotic cell death (159, 160). This metabolic plasticity necessitates the development of multi-targeted RCD induction strategies that concurrently inhibit multiple ferroptosis resistance axes to prevent adaptive escape (161). Furthermore, systemic induction of RCD can provoke excessive inflammation and collateral tissue damage, leading to adverse effects such as cytokine release syndrome or organ toxicity (162). These off-target effects highlight the critical need for tumor-specific activation of RCD inducers. One promising approach involves the design of prodrugs or gene-engineered therapies that are selectively activated within the tumor microenvironment, leveraging tumor-specific enzymatic activity or microenvironmental conditions to restrict RCD induction to malignant cells (163). For instance, tumor-targeted delivery of RCD inducers via nanocarriers responsive to tumor-specific stimuli can mitigate systemic toxicity while maximizing antitumor efficacy (164). Additionally, genetic engineering techniques, such as tumor-specific promoters driving expression of RCD-inducing genes, offer precise control over therapeutic activation (165). Addressing these challenges is essential to optimize the safety and effectiveness of combination therapies integrating RCD induction and immunotherapy in melanoma. Continued research into the molecular mechanisms underlying metabolic plasticity and off-target effects will inform the rational design of next-generation therapeutics with improved specificity and reduced adverse events (7, 166, 167). Ultimately, overcoming these hurdles will be pivotal to realizing the full clinical potential of RCD-based combination immunotherapies for melanoma patients. Collectively, the integrated therapeutic strategies targeting ferroptosis, pyroptosis, and necroptosis—including RCD inducers combined with ICIs, nanotechnology-driven precision delivery systems, and multi-targeted approaches to overcome metabolic plasticity and off-target toxicities—provide a comprehensive framework to enhance melanoma immunotherapy efficacy (**Figure 4**).

## 6 Clinical translation prospects and future research directions

With the growing understanding of the critical roles of regulated cell death (RCD) pathways in modulating antitumor immunity and overcoming immune checkpoint inhibitor (ICI) resistance, translating preclinical findings into effective clinical strategies has become a central focus in melanoma research (20). While substantial progress has been made in elucidating the molecular mechanisms underlying ferroptosis, pyroptosis, and necroptosis in melanoma progression and immunomodulation, bridging the gap between bench and bedside remains a key challenge (168). This section aims to summarize the current state of clinical translation of RCD-targeted combination therapies, highlight emerging biomarkers for patient stratification and treatment monitoring, explore novel therapeutic modalities that integrate RCD modulation with immunotherapy, and discuss the potential of personalized medicine and multi-pathway targeting to address melanoma heterogeneity (69). By synthesizing existing clinical evidence and outlining future research priorities, this section provides insights into how RCD-based strategies can be optimized to improve clinical outcomes for melanoma patients (14, 169).

### 6.1 Recent clinical trials targeting RCD pathways in melanoma and emerging evidence

Recent advances in clinical research have witnessed the initiation of multiple trials evaluating RCD-targeted therapies in melanoma, providing preliminary evidence for their safety, efficacy, and predictive biomarkers. These trials primarily focus on ferroptosis, pyroptosis, and necroptosis pathways, either as monotherapies or in combination with ICIs, addressing unmet needs in patients with advanced or ICI-refractory disease (113). For ferroptosis targeting, a phase I/II umbrella study evaluating multiple combination regimens in stage III/IV melanoma included a ferroptosis inducer combined with dual ICI therapy (nivolumab plus ipilimumab) (170). Among 110 enrolled patients, the ferroptosis-based combination achieved an objective response rate (ORR) of 31.8% in ICI-naive populations and 22.5% in ICI-refractory subgroups, with adverse events predominantly limited to grade 1–2 gastrointestinal discomfort and fatigue. Responders exhibited significantly elevated post-treatment serum lipid peroxidation (4-hydroxynonenal, 4-HNE) levels compared to non-responders, confirming on-target induction of ferroptosis. In pyroptosis-focused research, a phase I trial investigated an autophagy inhibitor (which indirectly enhances pyroptosis by promoting gasdermin E [GSDME] activation) in combination with nivolumab/ipilimumab in 42 patients with metastatic melanoma (171, 172). The trial reported a disease control rate (DCR) of 64.3%, with an ORR of 28.6% specifically in patients with high baseline intratumoral GSDME expression. Post-treatment tumor biopsies revealed increased intratumoral interleukin-1β (IL-1β) and interleukin-18 (IL-18) levels, indicating that pyroptosis activation correlates with therapeutic response. For necroptosis targeting, a phase I trial evaluating a SMAC mimetic (a necroptosis activator) combined with anti-PD-L1 antibody included 15 melanoma patients as part of a broader advanced solid tumor cohort (173). Subgroup analysis showed an ORR of 20% and a median progression-free survival (PFS) of 3.9 months in melanoma patients with positive intratumoral RIPK3 expression (IHC score ≥ 2+), while no objective responses were observed in RIPK3-negative patients, validating RIPK3 as a predictive biomarker for necroptosis-targeted therapy.

### 6.2 Barriers to clinical translation of RCD-targeted therapies in melanoma

Despite promising preliminary clinical results, several critical barriers hinder the successful translation of RCD-targeted therapies in melanoma, rooted in tumor-intrinsic characteristics and therapeutic limitations (114). Tumor metabolic plasticity represents a major challenge: melanoma cells can rapidly compensate for single RCD pathway inhibition (174). For example, induction of ferroptosis via GPX4 inhibition often triggers upregulation of ferroptosis suppressor protein 1 (FSP1)-mediated lipid peroxide detoxification, reducing long-term therapeutic efficacy (175, 176). Additionally, melanoma’s inherent intratumoral heterogeneity leads to variable RCD pathway sensitivity across tumor subpopulations, resulting in incomplete tumor eradication and increased recurrence risk (114, 177). The lack of standardized RCD-specific biomarkers further impedes clinical translation. Currently, no FDA-approved assays are available for quantifying key RCD pathway activation markers (such as GSDMD-N fragment for pyroptosis, phosphorylated MLKL for necroptosis) in clinical practice, making accurate patient stratification difficult (178, 179). Off-target toxicity also poses a significant hurdle. Systemic administration of RCD inducers may damage normal tissues with high metabolic activity or inherent RCD sensitivity: ferroptosis inducers can cause hepatotoxicity and renal tubular injury, while pyroptosis activators risk excessive release of pro-inflammatory cytokines (IL-1β/IL-18), potentially triggering systemic inflammatory response syndrome (179, 180).

### 6.3 Strategies to overcome clinical translation barriers

To address these challenges, targeted strategies are being developed to enhance the specificity, efficacy, and clinical applicability of RCD-targeted therapies in melanoma (14, 114). Multi-pathway combinatorial targeting, guided by multi-omics profiling, is a promising approach. Preclinical studies have shown that co-targeting ferroptosis and pyroptosis (e.g., GPX4 inhibitor + GSDMD activator) can overcome compensatory resistance by engaging complementary cell death mechanisms, amplifying DAMP release, and enhancing T cell infiltration (60, 181). Clinical trials are now evaluating this strategy in ICI-refractory melanoma, with a focus on dose de-escalation to minimize toxicity (14). Additionally, personalized medicine approaches leveraging genomic, transcriptomic, and metabolomic profiling can identify patient-specific RCD vulnerabilities (e.g., high GPX4 expression for ferroptosis targeting, low RIPK3 for necroptosis activation), enabling tailored combination regimens that maximize efficacy while reducing off-target effects (114, 182).

### 6.4 Insights from existing clinical studies and biomarker development

Recent clinical investigations into melanoma patients undergoing immune checkpoint inhibitor (ICI) therapy have revealed significant correlations between the expression of regulated cell death (RCD)-related genes within tumors and patient outcomes (14, 45). Specifically, genes such as GSDME, RIPK3, and GPX4, which are pivotal in pyroptosis, necroptosis, and ferroptosis respectively, exhibit distinct expression patterns that align with therapeutic response and survival duration (24, 82). For instance, elevated intratumoral expression of GSDME and RIPK3 has been associated with enhanced sensitivity to ICIs, likely due to their roles in promoting immunogenic cell death and subsequent activation of antitumor immunity (75, 183, 184). Conversely, GPX4, a key ferroptosis regulator, when overexpressed, may contribute to resistance by mitigating lipid peroxidation-induced cell death, thereby dampening immune-mediated tumor clearance. These findings underscore the potential utility of RCD-related gene expression profiles as predictive biomarkers for ICI responsiveness and prognosis in melanoma patients (14, 185).

Beyond tissue-based markers, dynamic changes in circulating damage-associated molecular patterns (DAMPs) linked to RCD, such as HMGB1 and cell-free DNA, have emerged as promising non-invasive biomarkers (186–188). Prospective clinical trials are warranted to validate whether fluctuations in these blood-borne molecules can serve as early indicators of response to combination therapies involving ICIs and agents targeting RCD pathways (189, 190). Such biomarkers could enable real-time monitoring of treatment efficacy, facilitating timely therapeutic adjustments. Moreover, integrating these circulating biomarkers with tumor gene expression data may enhance the precision of patient stratification and treatment personalization (191, 192).

However, current clinical studies also highlight challenges, including variability in biomarker expression due to tumor heterogeneity and the influence of the tumor microenvironment (TME) (193). The immunosuppressive milieu within melanoma can modulate RCD pathways and biomarker detectability, necessitating comprehensive analyses that consider both tumor-intrinsic and extrinsic factors (194). Additionally, the development of standardized assays and validation in large, diverse patient cohorts remain critical steps before clinical implementation (195, 196). Overall, existing clinical research provides compelling evidence that RCD-related molecular signatures and circulating DAMPs hold significant promise as predictive and monitoring biomarkers in melanoma immunotherapy, warranting further prospective validation (**Table 1**) (197, 198).

**Table 1:** Key preclinical and clinical findings of targeting ferroptosis, pyroptosis, and necroptosis in melanoma.

Pathway	Core molecular triggers	Key biomarkers	Representative therapeutic agents
Ferroptosis	Iron Overload; Lipid Peroxidation; GPX4 Inactivation; SLCA11/GSH Depletion; ACSL4 Upregulation	GPX4; SLC7A11; ACSL4; FTH1; 4-HNE; ROS	Erastin; RSL3; ML210; Sorafenib; Propafenone; Artesunate
Pyroptosis	Inflammasome-caspase-1/GSDMD;BRAF/MEK Inhibitors	GSDME; GSDMD; IL-1β; IL-18	Vemurafenib; Dabrafenib
Necroptosis	RIPK1/RIPK/MLKL Axis; TNF-α Inhibition; Epigenetic Silencing of RIPK3	RIPK3; p-MLKL; HMGB1	5-Azacytidine; Vorinostat; SMAC Mimetics

### 6.5 Exploration of novel therapeutic modalities

Innovative therapeutic strategies are increasingly focusing on harnessing regulated cell death (RCD) pathways to potentiate antitumor immunity in melanoma (14, 199). Oncolytic virus therapy represents a promising modality wherein viruses are engineered to selectively infect and lyse tumor cells (200). Recent advances have enabled the design of oncolytic viruses that co-express RCD activators, such as gasdermin D (GSDMD), a key executor of pyroptosis, alongside immune stimulatory molecules (70, 201). This dual expression facilitates not only direct tumor cell lysis but also the induction of robust immunogenic cell death, characterized by the release of pro-inflammatory cytokines and DAMPs that enhance dendritic cell activation and T cell priming (202). Preclinical models demonstrate that such engineered oncolytic viruses can reshape the tumor microenvironment (TME) towards an immune-activated state, thereby synergizing with ICIs to improve therapeutic efficacy (14, 203).

Adoptive cell therapies (ACT), including chimeric antigen receptor T-cell (CAR-T) therapy, have revolutionized hematologic malignancy treatment but face limitations in solid tumors like melanoma due to poor tumor infiltration and immunosuppressive TME (204, 205). Combining ACT with RCD inducers, particularly those triggering pyroptosis or ferroptosis, is an emerging approach to overcome these barriers (14, 206). Induction of pyroptosis within the TME can release inflammatory mediators that recruit and activate immune effector cells, enhancing CAR-T cell infiltration and function (207). Similarly, ferroptosis induction can modulate metabolic and oxidative stress pathways, disrupting tumor immune evasion mechanisms. Early-phase studies suggest that co-administration of RCD inducers with CAR-T cells may potentiate antitumor responses by remodeling the TME and overcoming resistance mechanisms inherent to melanoma (33, 208).

These novel therapeutic paradigms underscore the importance of integrating RCD pathway modulation with immunotherapy to achieve durable responses. Challenges remain in optimizing delivery systems, minimizing off-target effects, and identifying patient subsets most likely to benefit. Nonetheless, the convergence of oncolytic virotherapy and ACT with RCD activation represents a frontier in melanoma treatment, offering avenues to enhance immunogenicity and circumvent current therapeutic resistance (209, 210).

### 6.6 Personalized medicine and multi-pathway synergistic targeting

The heterogeneity of melanoma necessitates personalized therapeutic approaches tailored to the unique molecular and metabolic landscape of each patient’s tumor (211, 212). Integrative analyses encompassing genomic, transcriptomic, and metabolomic profiling enable the identification of the most susceptible regulated cell death (RCD) pathways within individual tumors (211, 213). For example, patients exhibiting high expression of ferroptosis-related genes such as GPX4 or ACSL4 may benefit from ferroptosis-inducing agents, whereas those with elevated pyroptosis or necroptosis signatures might respond better to therapies targeting gasdermin or RIPK kinase pathways (46, 214). This stratification facilitates the design of bespoke combination regimens that simultaneously or sequentially target multiple RCD pathways, maximizing tumor cell eradication while minimizing resistance (43, 209).

Crosstalk between RCD pathways, such as ferroptosis and pyroptosis, presents both challenges and opportunities (215, 216). Molecular interplay can lead to compensatory survival mechanisms if only a single pathway is targeted (20). Therefore, combinatorial strategies that concurrently induce ferroptosis and pyroptosis may produce synergistic antitumor effects by engaging distinct but complementary cell death mechanisms and enhancing immunogenicity (60, 217). Preclinical studies demonstrate that dual targeting can amplify DAMP release, promote antigen presentation, and stimulate robust adaptive immune responses, potentially overcoming immune escape and therapeutic resistance (14, 218).

Future research should focus on elucidating the mechanistic basis of RCD pathway interactions, optimizing timing and dosing of multi-pathway targeting agents, and developing predictive biomarkers to monitor pathway activation and therapeutic response (219). Clinical trials incorporating multi-omics-guided patient selection and combination RCD-targeted therapies are essential to validate these approaches. Ultimately, personalized medicine leveraging multi-pathway RCD modulation holds promise to transform melanoma treatment, achieving more potent, durable, and tailored immunotherapeutic outcomes (203, 220).

## 7 Conclusion

The exploration of regulated cell death (RCD) pathways—specifically ferroptosis, pyroptosis, and necroptosis—has significantly advanced our understanding of melanoma progression and the tumor immune microenvironment (7, 112). These non-apoptotic cell death mechanisms serve as pivotal modulators of tumor immunogenicity by facilitating the release of damage-associated molecular patterns (DAMPs) and pro-inflammatory cytokines (14, 221). This immunogenic cell death reshapes the typically immunosuppressive melanoma microenvironment, thereby enhancing anti-tumor immune responses (222, 223). From an expert perspective, the integration of these insights into therapeutic strategies represents a paradigm shift in melanoma treatment, moving beyond conventional apoptosis-centric approaches to harness the full spectrum of RCD pathways (8, 112).

The synergistic potential of targeting these immunogenic RCD pathways alongside immune checkpoint inhibitors (ICIs) is particularly noteworthy (112, 224). ICIs have revolutionized melanoma therapy but are often limited by primary or acquired resistance (124, 225). Inducing ferroptosis, pyroptosis, or necroptosis can potentiate the efficacy of ICIs by increasing tumor antigen presentation and promoting a pro-inflammatory milieu conducive to immune cell infiltration and activation (226, 227). This combinatorial approach addresses a critical unmet need by overcoming immune evasion mechanisms that tumors exploit (227). However, balancing the activation of these pathways to maximize therapeutic benefit while minimizing adverse effects remains a complex challenge. The metabolic plasticity of melanoma cells enables them to adapt to RCD induction, potentially undermining treatment efficacy (228). Moreover, the systemic induction of inflammatory cell death risks off-target toxicity, underscoring the necessity for precise spatial and temporal control of RCD activation.

Widespread and uncontrolled induction of ferroptosis, pyroptosis, and necroptosis also carries substantial safety risks that must be carefully weighed against therapeutic benefits (14, 229). Excessive RCD activation can trigger systemic inflammation due to uncontrolled release of DAMPs and pro−inflammatory cytokines, leading to cytokine release syndrome and immune-related adverse events (230–232). Normal tissues with high metabolic activity, iron dependency, or rapid turnover, including the liver, kidneys, heart, and intestinal mucosa, are vulnerable to off-target RCD−mediated tissue damage, which may cause organ dysfunction and limit clinical application (233–235). A comprehensive benefit–risk assessment is therefore essential for the safe translation of RCD−targeted strategies (14, 236).

To navigate these challenges, the development of tumor-targeted delivery systems is imperative. Nanotechnology-based platforms and gene-engineering techniques offer promising avenues to achieve selective induction of RCD within melanoma cells, thereby enhancing therapeutic index and reducing systemic toxicity. Concurrently, the identification and validation of predictive biomarkers are essential to stratify patients who are most likely to benefit from RCD-targeted therapies and to monitor treatment responses dynamically. Such biomarkers would enable personalized treatment regimens, optimizing efficacy and safety.

Future research must delve deeper into the intricate crosstalk among ferroptosis, pyroptosis, and necroptosis pathways within the melanoma microenvironment. Understanding how these pathways intersect and influence each other will inform the rational design of combination therapies that exploit their complementary mechanisms. Additionally, integrating advanced nanotechnologies and gene-editing tools can facilitate the development of precision medicine approaches that modulate multiple RCD pathways simultaneously. Translational efforts should prioritize clinical trials that evaluate these innovative combination therapies, with a focus on long-term outcomes and quality of life improvements for melanoma patients.

In conclusion, the strategic induction of immunogenic RCD pathways represents a transformative frontier in melanoma therapy. By leveraging the unique immunomodulatory properties of ferroptosis, pyroptosis, and necroptosis, and integrating these with existing immunotherapies, we can surmount current therapeutic resistance and achieve durable anti-tumor immunity. The path forward demands a multidisciplinary approach that harmonizes molecular biology, immunology, nanotechnology, and clinical oncology to refine these strategies. Ultimately, such concerted efforts hold the promise of fundamentally improving treatment efficacy and patient prognosis in melanoma, marking a significant milestone in the ongoing battle against this formidable malignancy.
